# Entropy for the Brain and Applied Computation

**DOI:** 10.3390/e23121639

**Published:** 2021-12-06

**Authors:** Roberto Zivieri, Israa Medlej, Ambra Fioravanti

**Affiliations:** 1 Istituto Nazionale di Alta Matematica (INdAM), Piazzale Aldo Moro 5, 00185 Rome, Italy; 2 Institute for Quantum Science and Engineering, Southern University of Science and Technology, Shenzhen 518055, China; medlej.israa@gmail.com; 3 Institute of Sciences and Technologies for Sustainable Energy and Mobility—CNR–STEMS, Via Canal Bianco 28, 44124 Ferrara, Italy; ambra.fioravanti@stems.cnr.it

Entropy is a quantity expressing the measure of disorder or unpredictability in a system, and, from a more general point of view, it can be regarded as an irreversible source of energy. Primarily, it was introduced as a classical thermodynamic quantity by Clausius and defined as the heat reversibly exchanged at a given temperature and has become a key concept in statistical physics because of the work of Boltzmann and Gibbs, and in information theory due to Shannon’s work; it takes different definitions that are all related to its statistical meaning [1]. Entropy also plays a pivotal role in chemistry, applied sciences, engineering and, last but not least, biological physics and biomedicine. Regarding biomedicine, for example, two decades ago, a method was introduced to calculate multiscale entropy for biological time series, enabling the separation between healthy and pathologic groups of individuals [2]. More recently, Prigogine’s minimum energy dissipation principle was theoretically proved in living systems by studying the rate of entropy production of irreversible reactions occurring in normal and cancer human cells, and its correlations with heat and matter transfer [3,4,5]. In strict connection with the above works dealing with entropy in biological systems, recently, the concept of entropy has also been applied to neuroscience, and its role in brain function has been highlighted as a paramount tool demonstrating the complex and intrinsic properties of the neural network system [6,7]. The description of the brain via the use of entropy has advanced three modern fields of research such as altered states of consciousness, the aging brain and the quantification of brain information processing, and their mutual interrelations [6]. A theory of conscious states has been developed by using neuroimaging research, and the concept of entropy was applied to the context of states of consciousness with special regard to the psychedelic state as a primary state of consciousness preceding the normal waking consciousness [8]. An understanding of the interplay between the brain region and the state of consciousness and the evolution of brain complexity, regarded as an entropy-enhancing process, has recently been proposed, leading to an increase in the space of states that can be visited, and to the accessibility of new channels [9]. Nowadays, nonlinear and complex system theories are considered promising candidates for analyzing the principles of operation of neural networks and their entropic content. The concept of computational entropy, for example, has been utilized in cryptographic primitives, namely, in cryptographic algorithms that are used to create cryptographic protocols in computer security systems. In most cases, computations involve hundreds of data points and have a high computational cost. However, the recent vectors with dissimilarity technique allows avoiding distance calculations of the most dissimilar vectors during computation, based on phase space reconstruction, leading to a reduction in the time needed to compute the sample entropy in random signals and electroencephalogram signals [10]. As a result, measurement of sample entropy computational time is accelerated. Moreover, the introduction of the concept of pseudo-entropy as a novel type of computational entropy that uses pseudorandom generators and is, in general, larger than the actual entropy has further advanced the field of brain entropy and computation.

The goal of this Special Issue is to investigate entropy-based novel methodologies to unravel, analyze and model intricate human brain networks and information flows. This Special Issue includes applied computational methods and theoretical methodologies from physics, biophysics, physical chemistry, electronics, engineering, biomedicine, biomathematics and neuroscience to provide additional insight into the role of the human brain based on entropy. 

This Editorial has the aim to attract submissions of theoretical and/or applied computation-oriented papers fitting within the topic of this Special Issue. 
